# MicroRNA-22 Controls Aberrant Neurogenesis and Changes in Neuronal Morphology After Status Epilepticus

**DOI:** 10.3389/fnmol.2018.00442

**Published:** 2018-12-11

**Authors:** Edward H. Beamer, Jeronimo Jurado-Arjona, Eva M. Jimenez-Mateos, James Morgan, Cristina R. Reschke, Aidan Kenny, Gioacchino de Leo, Luis A. Olivos-Oré, Marina Arribas-Blázquez, Stephen F. Madden, Jesús Merchán-Rubira, Norman Delanty, Michael A. Farrell, Donncha F. O’Brien, Jesus Avila, Miguel Diaz-Hernandez, M. Teresa Miras-Portugal, Antonio R. Artalejo, Felix Hernandez, David C. Henshall, Tobias Engel

**Affiliations:** ^1^Department of Physiology & Medical Physics, Royal College of Surgeons in Ireland, Dublin, Ireland; ^2^Institute of Physiological Chemistry, University Medical Center of the Johannes Gutenberg University Mainz, Mainz, Germany; ^3^Department of Molecular Biology, Centro de Biología Molecular “Severo Ochoa”, Consejo Superior de Investigaciones Científicas (CSIC), Universidad Autónoma de Madrid (UAM), Centro Investigación Biomédica en Red Enfermedades Neurodegenerativa (CIBERNED), Madrid, Spain; ^4^FutureNeuro Research Centre, RCSI, Dublin, Ireland; ^5^Department of Pharmacology and Toxicology, Veterinary Faculty, Instituto Universitario de Investigación en Neuroquímica, Universidad Complutense de Madrid, Madrid, Spain; ^6^Data Science Centre, Royal College of Surgeons in Ireland, Dublin, Ireland; ^7^Beaumont Hospital, Dublin, Ireland; ^8^Department of Biochemistry and Molecular Biology, Veterinary Faculty, Universidad Complutense de Madrid, Madrid, Spain

**Keywords:** status epilepticus, epilepsy, neurogenesis, microRNA-22, mouse model

## Abstract

Prolonged seizures (status epilepticus, SE) may drive hippocampal dysfunction and epileptogenesis, at least partly, through an elevation in neurogenesis, dysregulation of migration and aberrant dendritic arborization of newly-formed neurons. MicroRNA-22 was recently found to protect against the development of epileptic foci, but the mechanisms remain incompletely understood. Here, we investigated the contribution of microRNA-22 to SE-induced aberrant adult neurogenesis. SE was induced by intraamygdala microinjection of kainic acid (KA) to model unilateral hippocampal neuropathology in mice. MicroRNA-22 expression was suppressed using specific oligonucleotide inhibitors (antagomir-22) and newly-formed neurons were visualized using the thymidine analog iodo-deoxyuridine (IdU) and a green fluorescent protein (GFP)-expressing retrovirus to visualize the dendritic tree and synaptic spines. Using this approach, we quantified differences in the rate of neurogenesis and migration, the structure of the apical dendritic tree and density and morphology of dendritic spines in newly-formed neurons.SE resulted in an increased rate of hippocampal neurogenesis, including within the undamaged contralateral dentate gyrus (DG). Newly-formed neurons underwent aberrant migration, both within the granule cell layer and into ectopic sites. Inhibition of microRNA-22 exacerbated these changes. The dendritic diameter and the density and average volume of dendritic spines were unaffected by SE, but these parameters were all elevated in mice in which microRNA-22 was suppressed. MicroRNA-22 inhibition also reduced the length and complexity of the dendritic tree, independently of SE. These data indicate that microRNA-22 is an important regulator of morphogenesis of newly-formed neurons in adults and plays a role in supressing aberrant neurogenesis associated with SE.

## Introduction

Temporal lobe epilepsy (TLE) is the most common form of drug-refractory acquired epilepsy in adults (Ramey et al., 2013). TLE can emerge following an insult to the brain, such as traumatic brain injury or period of status epilepticus (SE; Pitkänen and Lukasiuk, 2009). A diverse set of molecular, cellular and structural changes are consistently found in the hippocampal formation of patients and animal models of TLE, which are implicated in the formation of a network with increased excitability and a lower threshold for seizure generation (Pitkänen and Lukasiuk, 2009). Amongst these epileptogenic processes are changes in receptor expression, the formation of aberrant synaptic connections, gliosis, selective cell loss and alterations to adult hippocampal neurogenesis (AHN; Pitkänen and Lukasiuk, 2009; Jessberger and Parent, 2015).

Data from rodent models indicate that, following SE, AHN increases sharply and remains elevated for up to 6 weeks (Parent et al., 1997; Gray and Sundstrom, 1998; Jessberger et al., 2007; Jessberger and Parent, 2015). Aberrant AHN not only involves the dysregulation of cell division and proliferation, but also of the maturation and morphology of newly-formed neurons (Kelly and Beck, 2017), their electrophysiological properties (Scharfman et al., 2000; Jakubs et al., 2006), migration (Han et al., 2016) and functional integration into existing neuronal circuits (Ge et al., 2007). This includes the aberrant migration of a subpopulation of newly-formed dentate granule cells into the dentate hilus, where they form a population of ectopic granule cells (Parent et al., 1997).

MicroRNAs (miRNA) are small non-coding RNAs that bind to target messenger RNA (mRNA) to reduce protein production via Argonaute (AGO)-mediated translational repression and other mechanisms (Bartel, 2004; Meister et al., 2004). MiRNAs have important effects on brain excitability and control several processes associated with epileptogenesis, including inflammation, synaptogenesis and ion channel expression (Jimenez-Mateos et al., 2015; Brennan and Henshall, 2018). Targeting key miRNAs using antisense oligonucleotide inhibitors (antagomirs) can alter seizure susceptibility, indicating the potential for miRNA-based therapeutics in epilepsy (Jimenez-Mateos and Henshall, 2013; Reschke and Henshall, 2015). A role for miRNAs has also been demonstrated in regulating each stage of the process of AHN, including proliferation, migration and dendritic arborization (Makeyev et al., 2007; Han et al., 2016; Santos et al., 2016; Mahmoudi and Cairns, 2017). Specific miRNAs also contribute to insult-induced neurogenic dysfunction. For example, miR-155 is necessary for inflammation-induced aberrant AHN (Woodbury et al., 2015). To our knowledge, however, no miRNAs have been specifically linked to the aberrant AHN that follows SE.

We recently screened AGO-bound miRNAs in the hippocampus of mice subjected to intraamygdala kainic acid (KA)-induced SE, identifying miR-22–3p (miR-22) as the most highly upregulated in the cell death spared, contralateral hippocampus (Jimenez-Mateos et al., 2015). Blocking miR-22, using antagomirs, resulted in exacerbation of subsequent spontaneous recurrent seizures (SRS) in mice, mediated in part by de-repression of the purinergic P2X7 receptor (Jimenez-Mateos et al., 2015; Engel et al., 2017). MiR-22, however, is likely to regulate other processes, besides inflammation (Xiong, 2012; Wang et al., 2017). Notably, miR-22 is also expressed in the neurogenic subgranular zone (Jimenez-Mateos et al., 2015) and has been implicated in controlling neurogenesis during development including lengthening of the cell cycle (Berenguer et al., 2013) and regulating cell migration (Volvert et al., 2014).

Here, we explored whether miR-22 regulates SE-induced aberrant AHN, focusing on the contribution of miR-22 to the regulation of the rate of adult neurogenesis, aberrant migration, dendritic arborization and the density and morphology of dendritic spines of newly-formed granule cells, both under normal conditions and following a period of KA-induced SE. Our results demonstrate that SE with a focal onset triggers aberrant adult neurogenesis, even in the contralateral dentate gyrus (DG) and that inhibition of miR-22 exacerbates multiple parameters including dendritic diameter and the density, volume and morphological maturity of dendritic spines. Further, dendritic length and complexity is unaffected by SE, but is modulated, independently of this insult, by miR-22.

## Materials and Methods

### Animal Model of Epilepsy

All animal procedures were performed in accordance with the principles of the European Communities Council Directive 2010/63/EU and National Institute of Health’s *Guide for the Care and Use of Laboratory Animals*. All studies involving animals were approved by the Research Ethics Committee of the Royal College of Surgeons in Ireland (REC 205 and 1322) and the Centro de Biología Molecular Severo Ochoa Institutional Animal Care and Utilization Committee (Comité de Ética de Experimentación Animal del CBM, CEEA-CBM), Madrid, Spain (PROEX293/15).

SE was induced in 10 week-old (20–25 g) adult male mice C57Bl/6 and *P2rx7*-green fluorescent protein (GFP) expressing mice [Tg(*P2rx7*-EGFP)FY174Gsat/Mmcd, stock 011959-UCD], from U.S. National Institutes of Health Mutant Mouse Regional Resource Centers and granted by Dr. M. Nedergaard (University of Rochester, Rochester, NY, USA) by unilateral stereotaxic microinjection of KA into the amygdala, as previously described (Moran et al., 2013). Briefly, deeply anesthetized mice (isoflurane 3%–5% induction and 1%–2% maintenance) were fitted with a guide cannula affixed over the dura using dental cement (coordinates from Bregma: AP= −0.94; ML= −2.85 mm) for the injection of KA. KA was injected via a 31-gauge internal cannula into the amygdala (0.3 μg in 0.2 μl vehicle; phosphate-buffered saline (PBS), pH adjusted to 7.4). Non-seizure control mice received 0.2 μl vehicle. Lorazepam (6 mg/kg, intraperitoneal) was administered 40 min following KA injection. Mice were euthanized at different time points following SE and brains perfused with PBS and 4% paraformaldehyde (PFA) for analysis.

### Antagomir Injection

MiR-22 inhibiting antagomirs (locked nucleic acid (LNA)- and 3′-cholesterol modified oligonucleotides; antagomir-22, Ant22) were administered via intra-cerebro-ventricular (i.c.v.) injection, as previously described (Jimenez-Mateos et al., 2015; Engel et al., 2017). Ant22 and a scrambled control (Scr) were purchased from Exiqon (Vadbaek, Denmark). The Ant22 sequence was CTTCAACTGGCAGCT and Scr sequence was ACGTCTATACGCCCA. Mice received 2 μl infusion of 0.5 nmol Ant22/Scr dissolved in PBS into the contralateral third ventricle 24 h prior to the induction of SE.

### Diaminobenzidine Staining and Immunohistochemistry

Diaminobenzidine (DAB) staining was carried out as previously reported (Engel et al., 2013). Mice were perfused with 4% PFA, brains post-fixed and cryoprotected in 30% sucrose solution, and 30 μm sagittal sections were cut on a Leica cryostat. Brain sections were then pre-treated for 1 h with 1% bovine serum albumin (BSA), 5% fetal bovine serum and 0.2% Triton™ X-100 followed by an overnight incubation with the primary antibody against the P2X7 receptor (1:400, Alomone, Jerusalem, Israel). Brain sections were then incubated in avidin–biotin complex using the Elite^®^ VECTASTAIN^®^ kit (Vector Laboratories Peterborough, UK). Chromogen reactions were performed with DAB and 0.003% hydrogen peroxide for approximately 10 min. Sections were coverslipped with FluorSave™.

Immunohistochemistry was performed as previously described (Engel et al., 2017). Briefly, 50 μm thick sagittal brain sections were cut on a Leica VT1200S vibratome, with series of every 8th section produced and stored at −20°C in glycoprotectant. Unless otherwise stated, procedures were carried out at room temperature. Sections were incubated in 0.1% Triton (in PBS) for 15 min, followed by 30 min in 1 M glycine, rinsed several times in PBS and incubated in 1% BSA for 45 min. Sections were again rinsed in PBS before being incubated overnight in primary antibody at 4°C. The next day, following washing in PBS, sections were incubated in secondary antibody for 2 h. Sections were then rinsed several times in PBS and incubated for 15 min in 4′,6-Diamidino-2-Phenylindole (DAPI, Calbiochem, San Diego, CA, USA) at a concentration of 1:500. Sections were again rinsed several times in PBS, rinsed in distilled water mounted and coverslipped with FluorSave™. The following primary antibodies were used: Goat anti-Doublecortin (anti-DCX; Santa Cruz Biotechnology, Dallas, TX, USA, 1:400), Mouse anti-NeuN (Merck Millipore, Tullagreen, Ireland, 1:400), Rabbit anti-GFAP (Cell Signaling, Danvers, MA, USA, 1:400) and Rabbit anti-GFP Molecular Probes (Invitrogen, Waltham, MA, USA, 1:400). Immunostaining with 5-iodo-2-deoxy-uridine (IdU) included a 30 min pretreatment with HCl 2N, followed by 30 min in borate buffer. The following secondary Alexa-conjugated antibodies were used at a final concentration of 1:400: Alexa 555-conjugated anti-goat; Alexa 555-conjugated anti-rabbit; Alexa 555-conjugated anti-mouse and Alexa 488-conjugated anti-rabbit (Life Technologies, Waltham, MA, USA).

### Western Blotting

Western blotting was performed as previously described (Engel et al., 2010). Thirty microgram of protein samples were boiled in gel-loading buffer and separated by sodium dodecyl sulfate polyacrylamide gel electrophoresis (SDS-PAGE). Proteins were transferred to nitrocellulose membranes and probed with the following primary antibodies: α-Tubulin (1:1,000, Sigma-Aldrich, Wicklow, Ireland) and P2X7 (1:400, Alomone, Jerusalem, Israel). Next, membranes were incubated with horseradish peroxidase-conjugated secondary antibodies (1:2,000, Isis Ltd, Bray, Ireland) and protein bands visualized using chemiluminescence (Pierce Biotechnology, Rockford, IL, USA). Gel bands were captured using a Fujifilm LAS-3000 (Fujifilm, Tokyo, Japan) and analyzed using Alpha-EaseFC4.0 software.

### Electrophysiological Recordings in Brain Slices

Electrophysiological recordings were carried out as previously described (Jimenez-Mateos et al., 2015). Briefly, 300 μm sagittal sections were cut using a vibratome (Integraslice 7550, Campden Instruments) from whole-brains from epileptic *P2rx7*-GFP expressing mice (14 days post-SE) and incubated in saline solution continuously bubbled with carbogen (95% O_2_/5% CO_2_) at room temperature for a maximum of 6 h before being transferred to the recording chamber. Slices were transferred to a submersion chamber attached to the stage of an upright microscope (Olympus BX51W1; UK), fixed with a nylon grid and superfused with a saline solution. Cells were viewed under a 63 × water immersion objective, with fluorescence illumination and a DL-604 OEM camera (Andor Technology, EU) used to visualize GFP-cells in the DG region of the hippocampus. Electrophysiological recordings were performed with an EPC10/2 patch-clamp amplifier using PatchMaster software (HEKA Electronic, Lambrecht, Germany). Patch pipettes had a final resistance of 5–6 MΩ when filled with a solution containing (in mM) 140 N-Methyl-D-glucamine (NMDG+), 5 EGTA, 3 MgCl_2_ and 10 HEPES (pH 7.2, adjusted with HCl; ≈290 mOsm). Membrane currents, measured in whole-cell configuration, were filtered at 3 kHz and sampled at 10 kHz. Once electrical access to the cytoplasm was established, cells were held at a voltage (V) of −70 mV. Series resistance (5–10 MΩ) was compensated by 80% and monitored throughout the experiment together with the cell membrane capacitance. Drug application was started at least 10 min after obtaining the whole-cell configuration, and experiments in which series resistance changed by more than 20% or holding current exceeded 20 pA were discarded. All recordings were obtained at room temperature. Currents were induced by 2′3′-O-(4-Benzoyl)benzoyladenosine 5′-triphosphat (BzATP; 100 μM; Sigma-Aldrich, Wicklow, Ireland) applied for 3 s at 2 min intervals onto the cell under investigation by means of a pneumatic drug ejection system (PDES-02DX, NPI Electronic GmbH, Germany). A-438079 (10 μM; Sigma-Aldrich, Wicklow, Ireland), a specific P2X7 antagonist, was applied from a separate glass pipette 2 min prior to, and during, agonist administration.

### MiRNA Extraction and Analysis

Total RNA was extracted using the Trizol method (Engel et al., 2013). The quality and quantity of RNA were measured using a Nanodrop Spectrophotometer (Thermo Scientific CA, USA) and samples with an absorbance ratio at 260/280 between 1.8 and 2.2 were considered acceptable. RNA dilutions were made up in nuclease-free water. For miRNA analysis, reverse transcription for individual qPCR was carried out using 500 ng of total RNA and the High-Capacity Reverse Transcription Kit (Applied Biosystems CA, USA). RT specific primers for miR-22 (ID: 000398. Thermo Fisher, CA, USA) were used for all miRNA reverse transcription. Individual qPCRs were carried out on the QuantStudio 12k Flex System (Thermo Fisher, CA, USA) using miR-22 specific Taqman microRNA assays (Thermo Fisher CA, USA). RNU6B (ID: 001093, Thermo Fisher CA, USA) was used for normalization of miRNA expression. A relative fold change in expression of the target gene transcript was determined using the comparative cycle threshold method (2^−ΔΔCT^).

### Temporal Lobe Epilepsy Patient Brain

This study was approved by the Ethics (Medical Research) Committee of Beaumont Hospital, Dublin (05/18), and written informed consent was obtained from the patient. The TLE patient used in our study has been referred for surgical resection of the temporal lobe for the treatment of intractable TLE. After temporal lobe resection, hippocampal tissue was obtained and frozen in liquid nitrogen and stored at −80°C until use. A pathologist assessed the hippocampal tissue and confirmed the presence of hippocampal sclerosis.

### *In situ* Hybridization

*In situ* hybridization was carried out as previously described (Jimenez-Mateos et al., 2012). Using RNAse free solutions, slides were treated with 0.25% acetic anhydride/0.1 M triethanolamine, followed by 5 μg/ml of proteinase K. Next, slides were rinsed in hybridization buffer. The probes to detect miR-22 or the positive control snRNA U6 were 2′-O, 4′-C methylene bicyclonucleoside monomer-containing oligonucleotides (LNA-modified; Qiagen, Vedbaek, Denmark). The nucleotide sequence for the anti-miR-22 probe was CTTCAACTGGCAGCTT/3Dig_N. A scrambled probe was also included to assess non-specific binding (Qiagen, Vedbaek, Denmark). Probes were incubated in 1:200 hybridization buffer overnight at 60°C. Sections were washed and then incubated with anti-DIG-PA antibody (1:1,000, Roche, Dublin. Ireland). The following day, sections were washed again and then reacted with color substrate solution (CSS: Nitroblue tetrazolium/BCIP stock solution; Roche, Dublin, Ireland). Slides were then rinsed, mounted and coverslipped with FluorSave™.

### Iodo-deoxyuridine Administration

Mice were injected with a single injection of IdU (42.75 mg/kg intraperitoneal; Sigma-Aldrich Arklow, Ireland) 72 h following SE. IdU dose used was based on an equimolar dose of 50 mg/kg 5-bromo-2′-deoxyuridine (BrdU).

### Cell Counting

To determine the number of P2X7-positive cells in the subgranular zone of the DG in Ant22 and Scr-treated epileptic mice, sagittal-cut brain slices were DAB-stained at the level of Bregma: AP = −1.94 mm. P2X7-positive cells were counted only within the dentate granular cell layer. Each count was the average of two adjacent slices. IdU was visualized via immunohistochemistry 6 weeks following SE as described above. Each count was the average of two adjacent slices (Bregma AP = −1.94 mm) including the dentate granular cell layer and the area of the hilus. The same counting procedure was used to calculate numbers for DCX-positive cells.

### Calculating Migration of Newly-formed Neurons

A common consequence of SE is dispersion of dentate granule cells (Murphy et al., 2012), leading to an increased width of the granular cell layer. This phenomenon has the potential to confound results for migration of newly-formed neurons, if using distance from the subgranular zone as the measurement. In order to control for this, we measured, not only distance from the subgranual zone (dSGZ), but also from the outer edge of the blade (dOE) and calculated migration as the percentage of granular cell layer width traversed by each cell, using the following calculation: dSGZ/(dSGZ+dOE)*100.

### Retroviral Stock Preparation

For labeling newly-formed neurons, a retroviral vector encoding postsynaptic density protein 95 (PSD95) fused to GFP (PSD95-GFP) was used. The vectors were provided by Prof. Lois (Department of Brain and Cognitive Science, Massachusetts Institute of Technology) and the methods for the generation of the vector have been described in detail elsewhere (Kelsch et al., 2008; Llorens-Martín et al., 2016). The PSD95-GFP retrovirus allows for visualization of dendritic spines under the green channel. Moreover, anti-GFP immunohistochemistry (red channel) allowed visualization of the whole neuronal morphology (Kelsch et al., 2008). Retroviral stocks were concentrated to working titers of 1 × 10^7^–2 × 10^8^ pfu/ml by ultracentrifugation. Since the retroviruses used are engineered to be replication incompetent, only dividing cells at the time of surgery can be infected (Kelsch et al., 2008). Four to six mice per experimental group were injected with the virus into the contralateral DG (coordinates from Bregma: AP:−2.0 mm, ML = 2.2 mm, DV 2.2 mm) using glass micropipettes, 1 week following SE. A total volume of 2 μl per contralateral DG of the viral suspension at a concentration of 10^7^–10^8^ pfu/ml was infused at 0.2 μl/min using an automatic infusion pump. Mice were killed 6 weeks following virus injection by intraperitoneal injection with pentobarbital and transcardially perfused with 0.9% saline solution followed by 4% PFA in PBS. Brains were removed and post-fixed overnight at 4°C in the same fixative.

### Morphometric Analysis

Three series of sections from each animal were used for the immunohistochemical detection of GFP. At least 50 randomly selected neurons from each experimental condition were reconstructed under a LSM710 Zeiss confocal microscope (25× oil immersion objective). Confocal stacks of images were obtained (Z-axis interval: 2 μm) and Z-projections were analyzed for the determination of total dendritic length and Sholl analysis. All cells were traced using the NeuronJ plugin for ImageJ software (ImageJ v.1.47, NIH, USA^1^). Sholl analysis was performed using the plugin Sholl Analysis for ImageJ. This analysis consists of placing a central point on the cell soma and tracing concentric circles (separated by a 50 μm distance interval). The number of times that the dendritic tree intersects with each circle is represented in the graphs (number of crossings). The Schoenen ramification index (SRI), which is the highest number of dendritic crossings at any single radius from the cell soma, was calculated as previously (Schoenen, 1982).

### Analysis of Dendritic Diameter

Dendritic trees were visualized in three dimensions using NeuronStudio (CNIC, Mount Sinai School of Medicine). Trees were traced using the in-built algorithm, detecting the intensity of fluorescence in three dimensions and corrected manually. Average diameter for each branch and for the entire tree of GFP-positive neurons were calculated.

### Calculation of Numbers of PSD95-GFP-Positive Clusters

PSD95-GFP-positive clusters were examined in each dendritic tree branching order. A minimum of 30 segments belonging to each experimental condition were analyzed for each branching order. Confocal stacks of images were obtained in a LSM710 Zeiss confocal microscope (63× oil immersion objective, XY dimensions: 67.4 μm; *Z*-axis interval: 0.13 μm). Dendritic trees were visualized in three dimensions using NeuronStudio. The dendritic length of each segment was measured (red channel, GFP), and the number, size (volume) and morphology was analyzed. Morphology of spines was assessed by the ratio of the head/neck. Spines with a head diameter greater than 0.4 μm and a head/neck ratio greater than 1.2 (Bourne and Harris, 2011) were considered “mushroom-shaped” spines.

### Data Analysis

**Figure 1:**
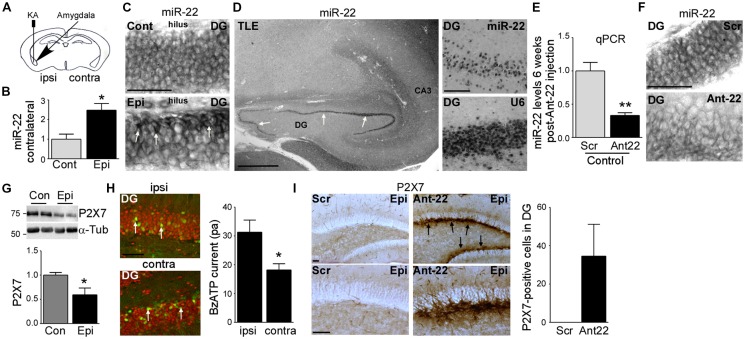
Increased miR-22 levels in the contralateral hippocampus during epilepsy in mice. **(A)** Schematic showing intraamygdala injection of kainic acid (KA) into the ipsilateral amygdala to induce status epilepticus (SE). **(B)** Graph showing increased levels of miR-22 in the contralateral dentate gyrus (DG) 2 weeks following SE (*n* = 4 per group, Student’s *t*-test: *t = 3.483, *p* = 0.0131). **(C)** *In situ* hybridization showing miR-22 in the DG of the contralateral hippocampus in non-epileptic control vehicle-injected mice and in mice subjected to SE. Of note, miR-22 levels seem to be mainly increased in the subgranular zone in the DG of the contralateral hippocampus during epilepsy (6 weeks following SE). Scale bar = 50 μm.** (D)** MiR-22 expression in the hippocampus in a patient suffering from temporal lobe epilepsy (TLE). MiR-22-positive cells were mainly localized to the DG (arrows and magnification). snRNA U6 was used as positive control. Scale bar = 1 mm. **(E)** Graph showing decreased miR-22 levels in the hippocampus (*n* = 4 per group Student’s *t*-test: **t = 4.955, *p* = 0.0026). **(F)** *In situ* hybridization showing decreased miR-22 levels in the subgranular zone of the DG 6 weeks following intra-cerebro-ventricular (i.c.v.) injection of Ant22. Scale bar = 50 μm. **(G)** Decreased P2X7 receptor expression in the contralateral hippocampus of epileptic mice 14 days following SE when compared to control mice (*n* = 4 per group, Students *t*-test: *t = 2.734, *p* = 0.034). **(H)** Lower BzATP-provoked currents detected by patch clamp from green fluorescent protein (GFP)-positive cells present in the contralateral DG of the hippocampus of epileptic *P2rx7*-GFP expressing mice when compared to GFP-positive cells from the ipsilateral DG 14 days following SE (*n* = 19 cells for ipsi- and 17 cells from contralateral hippocampus from nine mice per group, Student’s *t*-test, *t = 2.673, *p* = 0.0115). Scale bar = 50 μm. **(I)** Increased P2X7 receptor expression mainly localized to the subgranular zone of the DG of the contralateral hippocampus (arrows) in mice treated with Ant22 6 weeks following SE when compared to scramble-treated epileptic mice. *n* = 5, Scale bar = 50 μm.

All data are presented as mean ± standard error of the mean. Statistical analysis was performed using Graphpad Prism 5. Two group comparisons were made using unpaired Student’s *t*-test, while multi-group comparisons were made using two-way analysis of variance (ANOVA), with Tukey’s honest significant difference test (Tukey’s HSD) used for *post hoc* pairwise comparisons. Repeated measures two-way ANOVA was used to make comparisons between groups where a series of measurements have been taken from the same cell at different dendritic orders. In this case, *post hoc* pairwise comparisons were carried out using Bonferroni *post hoc* tests. Where groups contained a mean of zero (e.g., Figures 3E,G), these columns were removed from statistical analysis and Student’s *t*-test was used to make comparisons between non-zero columns. In Figure 1I, no statistical analysis was performed as one treatment group had a mean of zero.

**Figure 2:**
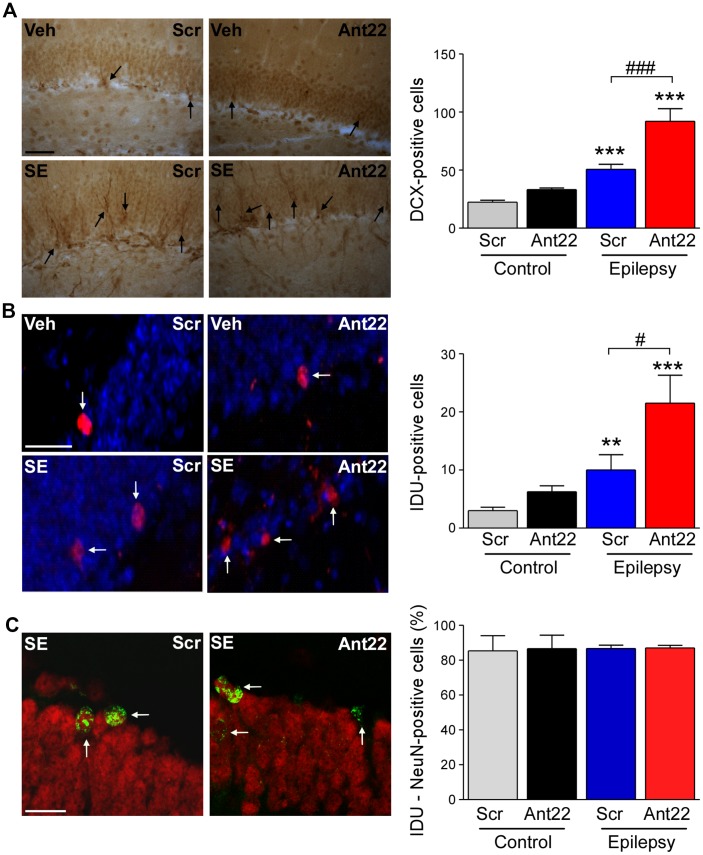
Increased neurogenesis 6 weeks following SE in mice with suppressed miR-22 expression. **(A)** Graph and representative images showing more Doublecortin-(DCX)-positive cells in the hippocampus of epileptic mice. DCX-positive cell numbers were even higher in epileptic mice treated with Ant22 when compared to epileptic mice treated with Scr (*n* = 3 (Scr Cont), 4 Ant22 (Cont and KA) and 6 (Scr KA), one-way ANOVA: *F*_(3,15)_ = 41.01, *p* < 0.0001; *post hoc* test (Bonferroni): ***t = 4.979, *p* < 0.001 (scramble/veh vs. scramble/KA); ***t = 9.726 (scramble/veh vs. antagomir/KA); ^###^*t* = 5.437, *p* < 0.001). Scale bar = 50 μm. **(B)** Graph and representative images showing more IdU-positive cells in epileptic mice 6 weeks following SE compared to control mice. The number of IdU-positive cells was also increased in epileptic mice treated with Ant22 when compared to Scr-treated epileptic mice (*n* = 3 (Scr Cont), 4 Ant22 (Cont and KA) and 6 (Scr KA), One-way ANOVA: *F*_(3,12)_ = 2.951 *p* < 0.0001 *post hoc* tests (multiple-comparisons Bonferroni): **t = 5.308, *p* < 0.01; ***t = 8.285, *p* < 0.001; ^#^*t* = 2.978, *p* < 0.05). Blue: nuclear marker DAPI, Red: IdU-positive cells. Scale bar = 50 μm. **(C)** Graph and representative image showing percentage of NeuN-positive IdU cells in the DG of the contralateral hippocampus 6 weeks following intraamygdala KA injection. Red: NeuN, Green: IdU (*n* = 3 (Scr Cont), 4 Ant22 (Cont and KA) and 6 (Scr KA). Scale bar = 20 μm.

**Figure 3:**
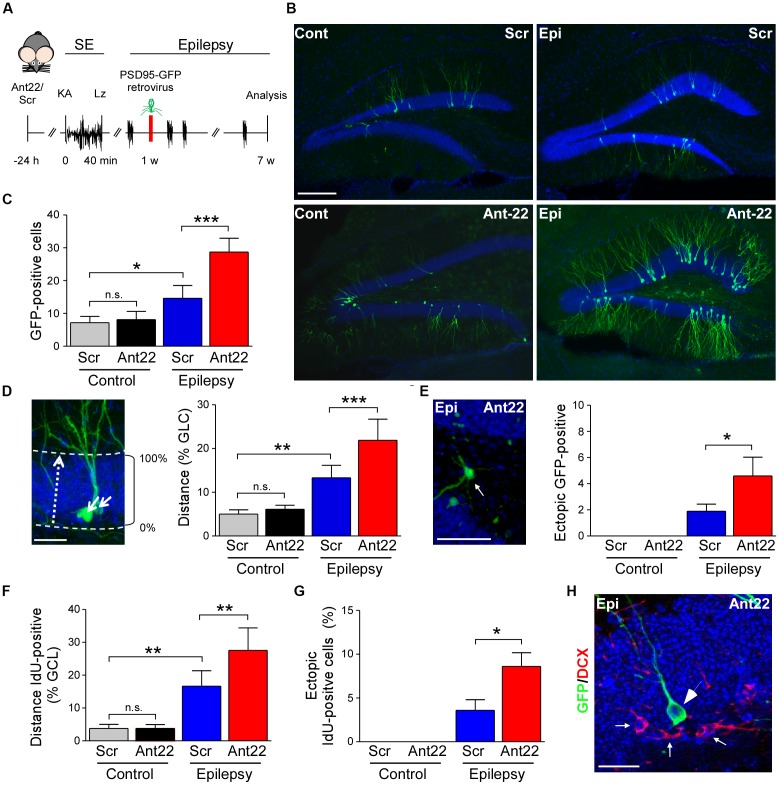
Viral transfection of postsynaptic density protein 95 (PSD95)-GFP of scramble and antagomir-22 treated mice following SE.** (A)** Schematic time-course of experimental design. Twenty four hours following i.c.v. injection of Ant22/Scr, mice were injected with intraamygdala KA to induce SE which was terminated with lorazepam (Lz). One week following SE, mice were injected into the contralateral DG with PSD95-GFP expressing retrovirus and brain analyzed 6 weeks later. **(B)** Representative images of the contralateral DG from Scr and Ant22-treated control and epileptic mice 6 weeks following transfection with PSD95-GFP. Virus was injected 1 week post-SE. Note, strong increase in GFP-positive cells in the contralateral DG in Ant22-treated epileptic mice. Scale bar = 200 μm. **(C)** Graph showing more PSD-95-GFP-positive cells in the contralateral DG in Ant22 treated mice (two-Way ANOVA: Scr vs. Ant22 (***F_(1,16)_ = 25.93, *p* < 0.001); Veh vs. KA (***F_(1,16)_ = 91.810, *p* < 0.001), interaction (***F_(1,16)_ = 20.28, *p* < 0.001). *Post hoc* tests (Tukey’s HSD): Veh/Scr vs. Veh/Ant22: n.s, *p* = 0.975; *Veh/Scr vs. KA/Scr: *p* = 0.012; ***KA/Scr vs. KA/Ant22: *p* < 0.001. **(D)** Representative image of migration calculation and graph showing mean (± SEM) distance traveled by PSD95-GFP-positive newly-formed neurons as percentage of width of granule cell layer (two-way ANOVA: Ant22 vs. Scr (**F_(1,16)_ = 15.94, *p* = 0.001); Veh vs. KA (***F_(1,16)_ = 94.88, *p* < 0.0001), interaction (**F_(1,16)_ = 9.65, *p* = 0.007). *Post hoc* tests (Tukey’s HSD): Veh/Scr vs. Veh/Ant22: n.s, *p* = 0.922; **Veh/Scr vs. KA/Scr: *p* = 0.001, ***KA/Scr vs. KA/Ant22: *p* < 0.001. **(E)** Example of PSD95-GFP-positive, newly-formed neuron in ectopic location in dentate hilus in an epileptic Ant22-treated mouse 6 weeks following transfection with PSD95-GFP virus and graph showing percentage of PSD95-GFP-positive, newly-formed neurons found ectopically in dentate hilus (Student’s *t*-test: KA/Scr vs. KA/Ant22: *t8 = 2.02, *p* = 0.039. **(F)** Graph showing increased distance traveled across the granule cell layer by IdU-positive neurons 6 weeks following SE (two-Way ANOVA: Scr vs. Ant22: *F_(1,16)_ = 8.32, *p* = 0.011; Veh vs. KA: ***F_(1,16)_ = 92.83, *p* < 0.001; Interaction: *F_(1,16)_ = 8.208, *p* = 0.011. *Post hoc* tests (Tukey’s HSD): Veh/Scr vs. Veh/Ant22: n.s, *p* = 1.000; **Veh/Scr vs. KA/Scr: *p* = 0.001; **KA/Scr vs. KA/Ant22: *p* = 0.004. **(G)** IdU-positive cells found ectopically in the contralateral hilus of the DG of epileptic Scr-treated mice 6 weeks following SE. Ectopic contralateral hilar cell count was exacerbated in epileptic Ant22-treated mice. Student’s *t*-test: KA/Scr vs. KA/Ant22: *t_8_ = 2.52, *p* = 0.018. **(H)** Representative image of PSD95-GFP-positive neurons in the contralateral DG of epileptic Ant22 mice 6 weeks following virus infection. Note, newly-formed neurons are all DCX-negative. DCX in red, DAPI in blue and PSD95-GFP transfected cells in green. Scale bar = 50 μm.

## Results

### Increased miR-22 Expression and Function in the Contralateral Hippocampus After Status Epilepticus

To elucidate the role of miR-22 in regulating AHN following SE, mice were given a unilateral microinjection of KA into the amygdala (Mouri et al., 2008; Figure 1A). In this model, prolonged seizures spread bi-laterally through the limbic system causing neurodegeneration in the ipsilateral but not contralateral hippocampus. The lesion is principally confined to the ipsilateral CA3 subfield. Epilepsy develops in all mice following a short latent period of 2–5 days with mice typically experiencing between 1–5 SRS per day (Mouri et al., 2008). We recently found miR-22 to be the most highly up-regulated miRNA in the contralateral hippocampus in the model and showed inhibition of this miRNA results in the generation of a secondary epileptogenic focus (Jimenez-Mateos et al., 2015).

First, we investigated miR-22 expression in the contralateral hippocampus during epilepsy development in the model. Levels of miR-22 were increased following SE in the DG (Jimenez-Mateos et al., 2015) and this elevation persisted into the time when SRS were actively occurring (Figure 1B). To identify the location of this upregulation we stained tissues sections for miR-22. *In situ* hybridization revealed that miR-22 was particularly evident in the subgranular zone of the DG corresponding to sites of enhanced neurogenesis after SE (Figure 1C). To determine whether miR-22 is also expressed in DG in epilepsy patients, we stained for miR-22 in hippocampal tissue resected from a drug-resistant TLE patient. This revealed miR-22 is highly expressed in the DG in human TLE (Figure 1D).

To investigate the functional contribution of miR-22 to AHN, we used a specific inhibitor of miR-22, antagomir-22 (Ant22). We have previously demonstrated that a single i.c.v. injection of 0.5 nmol of Ant22 reduces miR-22 levels in the hippocampus by over 70% for up to 2 weeks (Jimenez-Mateos et al., 2015). We now demonstrate by performing qPCR with hippocampal tissue from Scr-injected and Ant22-treated mice that this effect persists beyond this time point, with miR-22 expression suppressed for the full duration of this study (6 weeks; Figure 1E). *In situ* hybridization confirmed Ant22 reduced miR-22 expression in the DG and this lasted as long as 6 weeks following Ant22 administration (Figure 1F).

To extend the AGO-miRNA profiling evidence that miR-22 is functional in the contralateral hippocampus from epileptic mice, we measured changes to the purinergic P2X7 receptor, a validated miR-22 target (Jimenez-Mateos et al., 2015; Engel et al., 2017). Hippocampal protein expression of P2X7 was lower in the contralateral hippocampus of epileptic mice when compared to control mice (Figure 1G). To determine whether this decrease occurs in the subgranular zone, we visualized cells expressing the target *P2rx7* transcript using a transgenic mouse that expressed GFP driven by the P2X7 promoter (*P2rx7*-GFP; Engel et al., 2012). Whereas the GFP signal was evident in both the ipsi- and contralateral hippocampi of epileptic mice 2 weeks following SE, particularly in NeuN-positive neurons of the DG (Figure 1H), patch-clamp recordings demonstrated a stronger current induced by the P2X7 agonist BzATP in GFP-positive cells in the ipsilateral DG, when compared to the contralateral DG (Figure 1H). This suggests post-transcriptional repression of P2X7 in the contralateral hippocampus. In line with miR-22 targeting P2X7 during epilepsy (Engel et al., 2017), P2X7 protein levels in the contralateral hippocampus are higher in Ant22-treated epileptic mice when compared to epileptic Scr-treated mice, even 6 weeks following SE with this increase most evident in the subgranular zone of the DG (Figure 1I).

Taken together, our data reveal SE induces lasting upregulation and functional engagement of miR-22 in the contralateral hippocampus which is associated with a particular enrichment of miR-22 in the subgranular zone.

### Status Epilepticus-Induced Elevation in Neurogenesis Is Exacerbated by miR-22 Suppression

We have previously demonstrated that pre-treatment with Ant22 has no impact on the severity of SE (Jimenez-Mateos et al., 2015), however mice treated with Ant22 go on to develop a more severe epilepsy phenotype including the generation of a secondary, contralateral epileptic focus. The localization of miR-22 in the subgranular zone of the contralateral hippocampus following SE suggests that miR-22 may play a role in mediating SE-induced AHN. To investigate this, we studied the effects of miR-22 inhibition on neurogenesis following SE. Counts of DCX, a protein expressed in newly-formed migrating neuroblasts (von Bohlen und Halbach, 2007) revealed that 6 weeks following SE, DCX-positive cells were increased approximately 3-fold, compared to vehicle-injected non-seizure mice in the DG of the contralateral hippocampus (Figure 2A). DCX cell counts were approximately doubled in epileptic mice treated with Ant22 when compared to epileptic Scr-treated mice (Figure 2A). Inhibition of miR-22 in normal mice did not alter DCX counts (Figure 2A). These findings suggest that miR-22 may normally function to restrict the excess production of DCX-positive cells following SE.

To extend these findings, an additional set of mice were injected with a marker of dividing cells, IdU, 72 h following SE. Brains were then analyzed 6 weeks later. Supporting the DCX findings, the number of IdU-positive cells in the contralateral DG was elevated by approximately 75% in Scr-treated animals subject to SE 6 weeks earlier. IdU-labeling was approximately doubled in mice in which miR-22 was inhibited by Ant22 (Figure 2B). Ant22 did not affect IdU incorporation in non-seizure mice (Figure 2B).

To identify the phenotypes of these new-born cells, we counterstained IdU-labeled sections with the neuronal marker NeuN. This analysis revealed that the majority of IdU-positive cells were mature neurons (~80%). This distribution was similar between Scr and Ant22-treated mice indicating that miR-22 inhibition does not alter cell ontology (Figure 2C).

These findings indicate that miR-22 may act as a brake on excessive neurogenesis within the contralateral hippocampus in this model of focal-onset SE.

### MiR-22 Attenuates Neurogenesis and Aberrant Neuronal Migration in Mice Following Status Epilepticus

Our IdU findings suggest that most of the surviving newly-formed cells following SE become neurons. In order to investigate whether miR-22 influences the development of these neurons, we injected the contralateral DG of mice, 1 week following SE, with a retrovirus expressing GFP-tagged to the post-synaptic marker PSD-95 (Kelsch et al., 2008). GFP-expressing cells were identified in tissue sections using anti-GFP immunostaining 6 weeks later (Figure 3A). Analysis of GFP-positive cells revealed inhibition of miR-22 after SE induced an increase in AHN in mice (Figures 3B,C). Ant22 did not alter the number of GFP-positive cells in non-seizure control mice when compared to control Scr-injected mice (Figures 3B,C). Although viral infection is not considered a quantitative measure of neurogenesis, these findings are in line with our findings from DCX staining and IdU-labeling.

In order to measure whether miR-22 impacts on the migration of newly-formed neurons following SE, we measured migration of GFP-positive cells as a percentage of the width of the granule cell layer crossed from the subgranular zone (Figure 3D). In control mice, new neurons were only found within 5% of the inner edge of the granule cell layer. Ant22 treatment had no effect on migration in control mice, compared with Scr-treated mice (Figure 3D). Following SE, however, GFP-labeled neurons in Scr-treated mice had migrated across an average of 12% of the width of the granule cell layer. This SE-induced dysregulation of migration was exacerbated by Ant22 treatment, where the average migration of newly-formed neurons was 22% of the width of the granule cell layer (Figure 3D). This finding suggests that miR-22 functions to restrict aberrant migration of new-born neurons after SE.

SE also induced aberrant migration into the dentate hilus (Figure 3E). No ectopic hilar granule cells were found in non-seizure mice, whether treated with Scr or Ant22 (Figure 3E). Following SE, however, a small population of ectopic granule cells were found in the hilus in Scr-treated mice (approximately 2% of the total population of GFP-positive newly-formed neurons; Figure 3E). This was exacerbated in mice treated with Ant22, when compared with Scr-treated mice, with approximately 4.5% of GFP-positive, newly-formed neurons found in ectopic locations within the DG (Figure 3E). Confirming these results and excluding that the expression of GFP-retrovirus itself had an effect on migration of newly-formed neurons, the same results were obtained when analyzing IdU-positive cells with mice subjected to SE showing a dysregulated migration of newly-formed neurons which was exacerbated in Ant22-treated mice (Figure 3F). Ectopic IdU-positive neurons located in the hilar region were only found in epileptic mice, whether pre-treated with Scr or Ant22. Again, this was more pronounced in Ant22-treated mice (Figure 3G). All GFP-positive cells, whether in the granular cell layer or ectopically expressed, were DCX-negative (Figure 3H), confirming that this population of cells were identifiably mature neurons.

Together, these results suggest that miR-22 may function to oppose aberrant migration of new neurons in the hippocampus after SE.

### MicroRNA-22 Inhibition Reduces the Complexity of the Dendritic Tree

**Figure 4:**
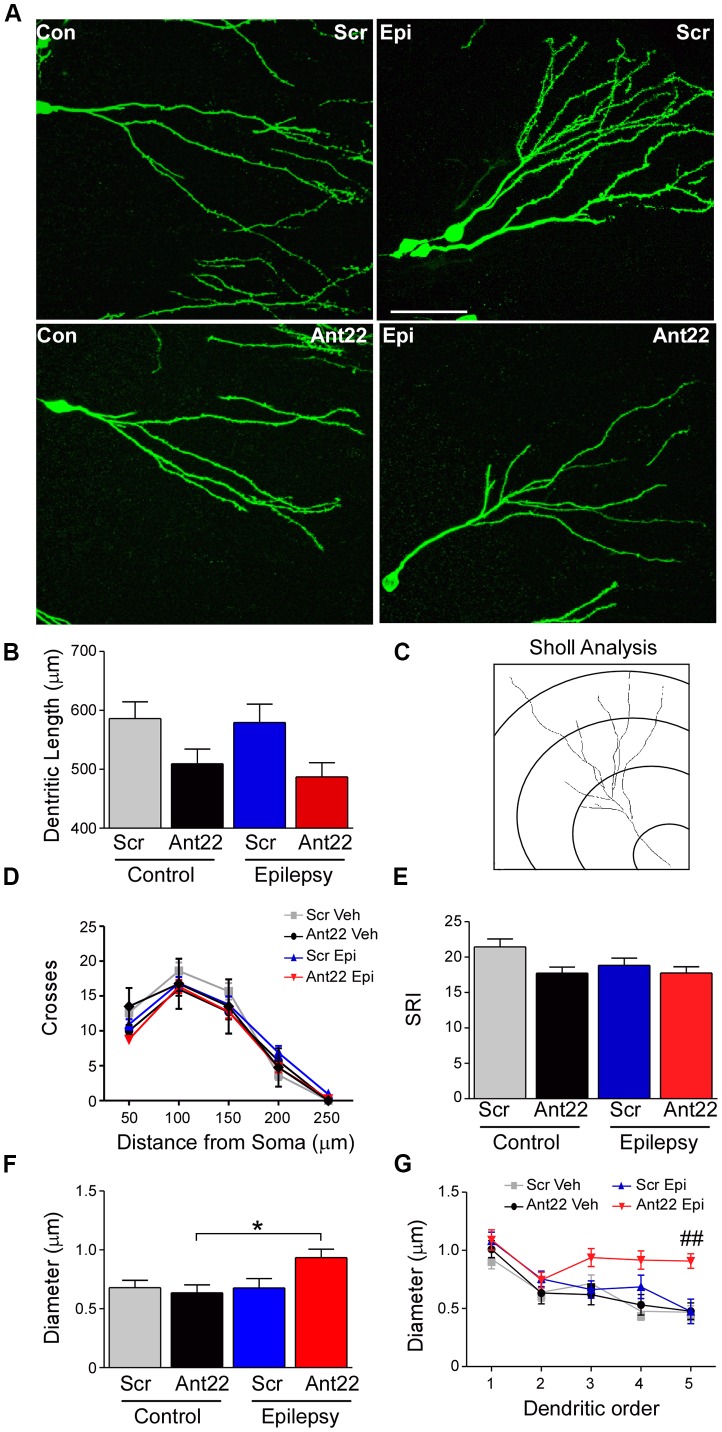
Ant22 reduces complexity of the dendritic tree, independently of SE. **(A)** Representative images of dendritic trees of PSD95-GFP-positive neurons of the four treatment groups (Scr and Ant22 control and epileptic mice 6 weeks following virus injection). Scale bar = 50 μm. **(B)** Dendritic length (combined length of entire dendritic tree) of all four treatment groups. Two-Way ANOVA: Scr vs. Ant22: *F*_(1,204)_ = 12.19, *p* < 0.001; Veh vs. KA: n.s, *F*_(1,204)_ = 0.97, *p* = 0.319; Interaction: n.s. *F*_(1,204)_ = 0.00, *p* = 986. *Post hoc* tests (Tukey’s HSD): Veh/Scr vs. Veh/Ant22: n.s. *p* = 0.066; Veh/Scr vs. KA/Scr: n.s. *p* = 0.867; KA/Scr vs. KA/Ant22: n.s. *p* = 0.070. **(C)** Cartoon illustrating Sholl analysis. Concentric circles at 50 μm intervals. Number of branches crossing at each interval are counted. **(D)** Sholl analysis of dendritic trees from four treatment groups. Branching was analyzed every 50 μm. **(E)** Schoenen Ramification Index (SRI) of dendritic trees from the four treatment groups showing decreased branching in GFP-positive cells in Ant22-treated mice. Two-way ANOVA: Scr. vs. Ant22: *F_(1,197)_ = 5.100, *p* = 0.025; Veh. vs. KA: *F*_(1,197)_ = 2.767, *p* = 0.098, n.s; Interaction: *F*_(1,197)_ = 1.54, *p* = 0.216, n.s). *Post hoc* tests (Tukey’s HSD): Veh/Scr vs. Veh/Ant22: n.s. *p* = 0.172; Veh/Scr vs. KA/Scr: n.s. *p* = 0.062; KA/Scr vs. KA/Ant22: n.s. *p* = 0.99. **(F)** Effect of Ant22-treatment and SE on dendritic diameter (two-Way ANOVA: Scr vs. Ant22: n.s. *F*_(1,16)_ = 2.19, *p* = 0.157; Veh vs. KA: n.s. *F*_(1,16)_ = 4.20, *p* = 0.0527; Interaction: n.s. *F*_(1,16)_ = 4.33, *p* = 0.054; *Post hoc* tests (Tukey’s HSD): Veh/Scr vs. Veh/Ant22: n.s. *p* = 0.977; Veh/Scr vs. KA/Scr: n.s. *p* = 1.000; KA/Scr vs. KA/Ant22: n.s. *p* = 0.095; *Veh/Ant22 vs. KA/Ant22: *p* = 0.044. **(G)** Graph showing increase in dendritic diameter in Ant22-treated mice following SE at higher dendritic orders (Repeated Measures Two-way ANOVA: F_3, 85_ = 14.45, *p* < 0.001; *post hoc* test (Bonferroni): ^##^Scr/KA vs. Ant22/KA, *t* = 3.882, *p* < 0.001).

We next analyzed the dendritic tree of PSD95-GFP-positive neurons in tissue sections from mice given Scr or Ant22. SE had no effect on dendritic length. Mir-22 inhibition, however, had a highly significant effect on dendritic length, although pair wise comparisons did not demonstrate a difference in either KA or vehicle-treated groups (Figures 4A,B). In line with these observations, a Sholl analysis (Figure 4C) indicated that inhibition of miR-22 leads to a shift of the branching distribution in newly-formed neurons (Figures 4D,E). Newly-formed granule neurons originating in a miR-22-deprived environment showed a decrease in the number of branches in the proximal segment of the neuron, represented by a decrease in the number of crossings close to the soma (Figures 4D,E). Induction of SE decreased the overall complexity of the dendritic tree (Figures 4D,E). Whilst there was a trend towards a decreased complexity associated with Ant22-treatment, this was not significant.

To further characterize the morphology of the dendritic tree, we measured the dendritic diameter in newly-formed neurons. Neither SE nor Ant22 alone had an effect on the diameter of dendrites in GFP-positive, newly-formed neurons. In Ant22-treated mice, however, SE induced a significant increase in dendritic diameter (Figure 4F). This was particularly pronounced at higher dendritic orders (Figure 4G).

Our results suggest a key role for miR-22 in controlling dendritic tree formation, with suppression of miR-22 resulting in reduced dendritic growth and altering branching of the dendritic tree even in the absence of a pathological insult. A further effect on diameter of dendrites can be observed when miR-22 is inhibited in mice previously subjected to SE.

### MicroRNA-22 Inhibition Increases Spine Density and Spine Volume in Newly-Formed Neurons Following Status Epilepticus

**Figure 5:**
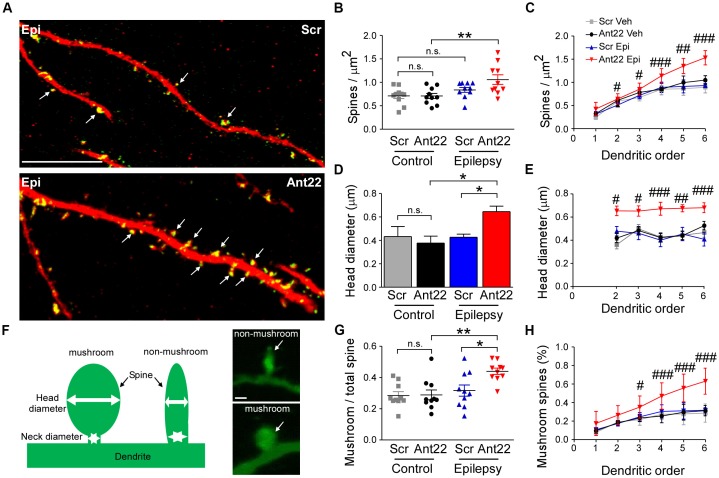
MiR-22 inhibition increases spine density and volume on dendrites of newly-formed neurons following SE. **(A)** Representative images showing increased spine density in Ant22-treated epileptic mice when compared to *Scr*-treated epileptic mice. Dendrites are shown in red and spines in green (arrows). Scale bar = 20 μm. **(B)** Graph showing that SE increases spine density and that this is exacerbated by Ant22 treatment (two-way ANOVA: Scr vs. Ant22: n.s. *F*_(1,36)_ = 2.34, *p* = 0.135; Veh vs. KA: *F*_(1,36)_ = 11.26, *p* = 0.002; Interaction: n.s. *F*_(1,36)_ = 2.47, *p* = 0.125). *Post hoc* tests (Tukey’s HSD): Veh/Scr vs. Veh/Ant22: n.s. *p* = 1.000; Veh/Scr vs. KA/Scr: n.s. *p* = 0.593; KA/Scr vs. KA/Ant22: n.s. *p* = 0.144; **Veh/Ant22 vs. KA/Ant22: *p* = 0.007. **(C)** Elevated spine density in Ant22-treatd mice following SE most pronounced at higher dendritic orders: Repeated-measures ANOVA: *F*_(3,198)_ = 92.16, *p* < 0.0001, *post hoc* test (Bonferroni; scramble KA vs. antagomir KA): ^#^*t* = 2.840, *p* < 0.05 (*order ii*); ^#^*t* = 3.112 (*order iii*), *p* < 0.05; ^###^*t* = 4.384, *p* < 0.001 (*order iv*); ^##^*t* = 3.069, *p* < 0.01 (*order v*); ^###^*t* = 4.421, *p* < 0.001 (*order vi*). **(D)** Graph showing average spine volume increased following KA-induced SE in Ant22-treated mice, compared with Scr-treated controls (two-Way ANOVA: Scr vs. Ant22: *F*_(1,26)_ = 5.87 *p* = 0.023; Veh vs. KA: n.s. *F*_(1,26)_ = 2.933, *p* = 0.099; Interaction: *F*_(1,26)_ = 5.66, *p* = 0.025. *Post hoc* tests (Tukey’s HSD): Veh/Scr vs. Veh/Ant22: n.s. *p* = 1.000; Veh/Scr vs. KA/Scr: n.s. *p* = 0.910; *KA/Scr vs. KA/Ant22: *p* = 0.011; *Veh/Ant22 vs. KA/Ant22: *p* = 0.048. **(E)** Increased spine volume in KA and Ant22-treated mice consistent across all dendritic orders (Repeated Measures ANOVA: *F*_(3, 80)_ = 32.26, *p* < 0.0001; *post hoc* test (Bonferroni; scramble KA vs. antagomir/KA): ^#^*t* = 2.840, *p* < 0.05 (*order ii*); ^#^*t* = 3.112, *p* < 0.05 (*order iii*); ^###^*t* = 4.384, *p* < 0.001 (*order iv*); ^##^*t* = 3.609, *p* < 0.01 (*order v*); ^###^*t* = 4.421, *p* < 0.001 (*order vi*)). **(F)** Examples of mushroom-shaped and non-mushroom shaped spines. Spines with a head diameter greater than 0.4 μm and a head/neck ratio greater than 1.2 (Bourne and Harris, 2011) were considered “mushroom-shaped.” Scale bar = 0.5 μm. **(G)** Ratio of mushroom/non-mushroom shaped spines increased following KA-induced SE in Ant22-treated mice, compared with *Scr*-treated controls (two-way ANOVA: Scr vs. Ant22: *F*_(1,36)_ = 4.95, *p* = 0.032; Veh vs. KA: *F*_(1,36)_ = 10.22, *p* = 0.003; Interaction: *F*_(1,36)_ = 4.35, *p* = 0.044. *Post hoc* tests (Tukey’s HSD): Veh/Scr vs. Veh/Ant22: n.s. *p* = 1.000; Veh/Scr vs. KA/Scr: n.s. *p* = 0.861; *KA/Scr vs. KA/Ant22: *p* = 0.021; **Veh/Ant22 vs. KA/Ant22: *p* = 0.003. **(H)** Increased density of mushroom-shaped spines in mice following treatment with Ant22 and 6 weeks following KA-induced SE most marked at higher dendritic orders (Repeated Measures ANOVA: *F*_(3,198)_ = 59.24, *p* < 0.0001; *post hoc* test (Bonferroni; scramble/KA vs. antagomir/KA): ^#^*t* = 2.827, *p* < 0.05 (*order iii*); ^###^*t* = 4.351, *p* < 0.001 (*order iv*); ^###^*t* = 6.069, *p* < 0.001 (*order v*); ^###^*t* = 7.737, *p* < 0.001 (*order vi*)).

We next investigated dendritic spine density and volume in GFP-labeled new-born neurons, which are important contact points for excitatory synaptic input and associated with excitability and may be altered in epileptogenesis (Wong and Guo, 2013). Inhibition of miR-22 had no effects on the density of dendritic spines in GFP-labeled cells in non-seizure mice (Figures 5A,B). The combination of SE and Ant22 treatment, however, led to an elevation in spine density (Figures 5A,B), particularly at higher dendritic orders (Figure 5C). Not only did miR-22 suppression in combination with SE increase the number of dendritic spines, but the average volume of the spines was also increased (Figure 5D). Unlike with spine density, however, this change was consistent across dendritic orders (Figure 5E).

Mushroom-shaped spines (Figure 5F) are indicative of a fully mature spine and these spines tend to have an increased density of neurotransmitter receptors and therefore more efficiently translate inputs into an action potential (Sultan et al., 2015). The ratio of mushroom/non-mushroom-like spines was unaffected by Ant22 in control mice, but was increased following SE, and this SE-induced effect was exacerbated in the presence of Ant22 (Figure 5G). The increased percentage of mushroom spines in SE Ant22-treated mice was most pronounced at higher dendritic orders (Figure 5H). Inhibition of miR-22 after SE therefore leads to an increase in spine density, spine volume and a mushroom-like morphology on newly-formed neurons possibly contributing to an aggravated epileptic phenotype.

## Discussion

The present study is the first reported involvement of miRNAs in mediating SE-induced aberrant AHN. An increasing number of studies have demonstrated that dysregulation of the miRNA profile is both a consequence of, and functional contributor to epileptogenesis (Gorter et al., 2014; Brennan and Henshall, 2018) and miRNAs have also been implicated in each step of neurogenesis (Makeyev et al., 2007; Han et al., 2016; Santos et al., 2016; Mahmoudi and Cairns, 2017). A contribution of miRNA to mediating the aberrant AHN initiated by SE, however, has not, until now, been established. We have previously demonstrated that miR-22 functions as an endogenous antiepileptogenic agent, supressing the establishment of a contralateral epileptic focus (Jimenez-Mateos et al., 2015). Here, we demonstrate that miR-22 upregulation in the neurogenic subgranular zone of the DG persists for weeks following SE, where it acts to put a brake on aberrant AHN. Suppression of miR-22, prior to the induction of SE, exacerbates aberrant AHN in the contralateral hippocampus of mice. This effect is not limited to the number of new neurons produced, but also to their migratory path, dendritic morphology and the density, volume and morphological maturity of their dendritic spines.

It is now well established that experimentally-induced SE leads to a sustained period of elevated AHN in the DG of rodents (Parent et al., 1997; Gray and Sundstrom, 1998; Jessberger et al., 2007; Jessberger and Parent, 2015). To our knowledge, however, this is the first demonstration of bilateral aberrant AHN, following unilateral injection of a chemoconvulsant, suggesting that SE with a focal onset may promote aberrant AHN at distal locations. While this is an important finding in itself, it has also allowed us to investigate neurogenesis in the contralateral DG, in the absence of the potentially confounding effects of wide-scale neuronal death (Mouri et al., 2008).

The principal finding of this study is that a unilateral injection of KA induces an increased rate of neurogenesis in the contralateral hippocampus and that this aberrant AHN is exacerbated by miR-22 inhibition. Previous reports of miR-22 involvement in neurogenesis are scarce, however Berenguer et al. (2013) report that this miRNA lengthens the cell cycle and suppresses cerebellar granule neuron precursor proliferation during development. We also demonstrate that miR-22 plays a role in maintaining the integrity of migratory pathways and reducing the number of newly-formed neurons located ectopically in the hilus and reducing the average distance traveled by these cells across the granule cell layer. Aberrant migration of new neurons following SE in rodent models is well-documented (Jessberger et al., 2005, 2007; Walter et al., 2007), however, this is the first report in the contralateral DG following unilateral chemoconvulsant injection. We did not establish the mechanism through which miR-22 controls migration, however, DCX, a microtubule-interacting protein associated with the migration of immature neurons (Tang et al., 2014), is a target of miR-22 (von Bohlen und Halbach, 2007) via components of the CoREST/REST transcriptional repressor complex (Volvert et al., 2014).

Furthermore, we report that while SE had no impact on the length of branching of the dendritic tree, miR-22 inhibition reduced dendritic length, independently of KA treatment. This suggests that miR-22, besides its role in suppressing aberrant AHN, also plays a role in dendritic arborization under normal conditions. A major finding in the present study was that miR-22 inhibition combined with SE led to a marked increase in dendritic diameter. No change in dendritic diameter of newly-formed neurons was found to result from SE in Scr-treated mice, in contrast to the findings of Arisi and Garcia-Cairasco (Arisi and Garcia-Cairasco, 2007), who reported a decrease in dendritic diameter in a pilocarpine rat model.

Neither SE nor miR-22 inhibition in isolation had any effect on spine density, volume or morphological maturity. MiR-22 inhibition and SE together, however, increased the density of spines, spine volume and an increased percentage of spines adopting a mature “mushroom-like” morphology. This suggests that miR-22 is responsible for suppressing SE-driven alterations in growth and shape of dendritic spines. Whether this effect is restricted to newly-formed neurons or reflects plastic changes in the granule cell population at large, is unclear. In contrast to our findings, most authors have reported a reduction in spine density following SE (Murphy and Danzer, 2011). Isokawa (Isokawa, 2000), however, demonstrated that this reduction is short-lived in the pilocarpine rat model and within 35 days, spine density was significantly higher than normal levels. Our finding that SE alone had no effect on spine density may therefore be a specific effect of the time-point analyzed. Murphy et al. (2011) suggest that neurons born shortly after SE include two subpopulations; one with a decreased number of spines and one with an increased number of spines. An alternative explanation for the pro-spinogenic effect of miR-22 is that it is involved in selective targeting of one subpopulation for phagocytosis and that the apparent increase in average spine density, in fact represents the selective sparing of the second subpopulation. This may also offer an alternative explanation for changes seen in dendritic diameter.

While our results demonstrate that inhibition of miR-22 exacerbates aberrant AHN, we have not established the mechanism through which this occurs. Neuroinflammation supresses neurogenesis in most cases, but it has become clear that this relationship is more complicated than previously thought (Ekdahl et al., 2009) and in the post-SE DG, inflammation may be pro-neurogenic (Mishra et al., 2015). We have previously demonstrated that P2X7, a key regulator of neuroinflammation, is a target of miR-22 (Jimenez-Mateos et al., 2015). It is possible that miR-22 control of AHN may be secondary to control of inflammatory pathways via P2X7. Further, while the severity of SE is unchanged following Ant22 injection, the frequency and severity of spontaneous epileptic seizures is increased (Jimenez-Mateos et al., 2015). This may be related to network level changes associated with aberrant AHN. This increase in seizure severity, however, may also contribute to changes in AHN.

The majority position is that an expansion in neurogenic activity and dysregulation of migration of newly-formed neurons contributes to network hyperexcitability and epileptogenesis (Jung et al., 2004; Scharfman and Pierce, 2012; Cho et al., 2015) and underlies comorbidities, by disrupting the normal functioning of hippocampal networks (Myers et al., 2013). Causality, however, is difficult to establish and some authors have proposed that these processes, in fact, serve to repair the injured brain (Sun et al., 2007), with hilar ectopic granule cells contributing to inhibitory drive (Lacefield et al., 2012). Others consider aberrant AHN an epiphenomena, offering no contribution to epileptogenesis (Kondratiuk et al., 2015; Neuberger et al., 2017; Zhu et al., 2018), while the long-term costs incurred by the depletion of the neurogenic pool, including an increased susceptibility to seizures, may be of greater consequence (Sierra et al., 2015). While most of the evidence from rodent models suggests an instrumental role for aberrant AHN in epileptogenesis (Cho et al., 2015), some doubts have been raised regarding the relevance of AHN to humans, with Sorrells et al. (2018) reporting no AHN detectable above age 13 (Sorrells et al., 2018). This potentially calls into question the relevance of findings from rodent models of disease. The matter remains, however, highly contentious, with subsequent reports of persistent human AHN (Boldrini et al., 2018) and disagreements over the interpretation of cellular markers. Further, evidence suggests a dissociation between age-related decline in basal rates of AHN and status-epilepticus induced elevations, at least in rodents (Gray and Sundstrom, 1998). This suggests that, even in the case that AHN does not occur in humans under normal conditions, insult-induced aberrant AHN is likely still of clinical relevance. This is supported by consistent clinical findings of hilar ectopic granule cells in tissue resected from TLE patients, suggestive of a period of aberrant AHN (Althaus et al., 2015). MiR-22 may be involved in modulating SE-driven dendritic morphogenesis and spinogenesis on newly-formed neurons, or, alternatively, the observed effects may be the consequence of miR-22 control over differential survival. Either way, the consequences of increased dendritic diameter are likely to include increased efficiency in synaptic integration, larger post-synaptic currents and ultimately, an increase in neuronal excitability (Magee, 2000). While dendritic spines are associated with excitatory synapses and the increased density, volume and morphological maturity may all contribute towards an increase in excitatory drive, transmission and fidelity (Araya et al., 2006). The increased excitability of individual neurons is likely to translate into network-wide hyperexcitability and a reduced threshold for ictogenesis.

## Conclusion

The present study is the first to demonstrate a role for miRNAs in mediating SE-driven expansion in neurogenic activity. We have previously demonstrated that miR-22 supresses the emergence of a secondary, contralateral epileptic focus, through modulation of neuroinflammatory pathways via suppression of P2X7, thereby acting as an endogenous antiepileptogenic agent (Jimenez-Mateos et al., 2015). Here, we report that miR-22 acts as a brake on aberrant AHN, adding to the evidence that miR-22 plays a multi-faceted role in supressing the development of TLE and suggesting that it may represent an attractive candidate for designing a disease-modifying intervention.

## Author Contributions

EB performed immunohistochemistry, contributed to data analysis and wrote the article. JJ-A performed injections of viral vectors, contributed to data analysis and edited the manuscript. EJ-M and AK performed *in situ* hybridization. JM and JM-R performed immunohistochemistry. CR performed immunohistochemistry and contributed to data analysis. GL carried out P2X7 immunohistochemistry. LO-O and MA-B performed patch-clamp recordings. SM performed statistical analysis. ND, MF, JJ-A, MM-P, AA and JA edited the manuscript. DO’B provided human brain sample. MD-H edited the manuscript and helped with virus injection. FH provided antibodies and edited the manuscript. DH wrote parts of the manuscript and edited the manuscript. TE performed KA injections and wrote the manuscript.

## Conflict of Interest Statement

The authors declare that the research was conducted in the absence of any commercial or financial relationships that could be construed as a potential conflict of interest.

## Footnotes

^1^http://rsbweb.nih.gov/ij/
